# Factors Associated with Opioid Use in a Cohort of Patients Presenting for Surgery

**DOI:** 10.1155/2015/829696

**Published:** 2015-12-31

**Authors:** Jennifer M. Hah, Yasamin Sharifzadeh, Bing M. Wang, Matthew J. Gillespie, Stuart B. Goodman, Sean C. Mackey, Ian R. Carroll

**Affiliations:** ^1^Division of Pain Medicine, Department of Anesthesiology, Perioperative, and Pain Medicine, Stanford University, Palo Alto, CA, USA; ^2^Stanford Systems Neuroscience and Pain Lab (SNAPL), Stanford University, Palo Alto, CA, USA; ^3^Department of Orthopaedic Surgery, Stanford University, Palo Alto, CA, USA

## Abstract

*Objectives*. Patients taking opioids prior to surgery experience prolonged postoperative opioid use, worse clinical outcomes, increased pain, and more postoperative complications. We aimed to compare preoperative opioid users to their opioid naïve counterparts to identify differences in baseline characteristics.* Methods*. 107 patients presenting for thoracotomy, total knee replacement, total hip replacement, radical mastectomy, and lumpectomy were investigated in a cross-sectional study to characterize the associations between measures of pain, substance use, abuse, addiction, sleep, and psychological measures (depressive symptoms, Posttraumatic Stress Disorder symptoms, somatic fear and anxiety, and fear of pain) with opioid use.* Results*. Every 9-point increase in the Screener and Opioid Assessment for Patients with Pain-Revised (SOAPP-R) score was associated with 2.37 (95% CI 1.29–4.32) increased odds of preoperative opioid use (*p* = 0.0005). The SOAPP-R score was also associated with 3.02 (95% CI 1.36–6.70) increased odds of illicit preoperative opioid use (*p* = 0.007). Also, every 4-point increase in baseline pain at the future surgical site was associated with 2.85 (95% CI 1.12–7.27) increased odds of legitimate preoperative opioid use (*p* = 0.03).* Discussion*. Patients presenting with preoperative opioid use have higher SOAPP-R scores potentially indicating an increased risk for opioid misuse after surgery. In addition, legitimate preoperative opioid use is associated with preexisting pain.

## 1. Introduction

Previous research has reported prolonged postoperative opioid use in patients already taking opioids prior to surgery [1–3]. A small retrospective study of patients undergoing orthopedic surgery for trauma reported that positive toxicology results at the time of injury predicted an extended duration of postoperative prescription opioid use [1]. Similarly, 30% of women taking preoperative opioids were taking them 6 months after gynecologic surgery compared to 2.2% of women not taking preoperative opioids [2]. These findings extend to patients with preoperative chronic opioid use. 77% of chronic opioid users undergoing bariatric surgery continued to use opioids in the year after surgery [4]. Likewise, a higher proportion of chronic opioid users compared to nonusers continued to take opioids 58 months after total hip arthroplasty [5]. Furthermore, compared to their opioid naïve counterparts, preoperative prescription opioid users have worse functional outcomes [3], worse clinical outcomes [5, 6], increased hospital length of stay [5, 6], increased pain severity after surgery [7] even in the context of increased opioid requirements [8], increased specialist referrals for pain management [6], more dissatisfaction with the surgical outcome [7], and a higher prevalence of postsurgical complications [6]. Particularly notable is the increased mortality associated with preoperative chronic opioid use. A history of chronic opioid use in patients with end-stage renal disease was associated with 1.6- to 2-fold increased risk of death after kidney transplantation [9].

Mechanisms for reduced opioid cessation after surgery in patients taking preoperative opioids are unclear. Research suggests a decreased resolution of postoperative allodynia [10]. In mice, morphine leads to enhanced nociceptive sensitivity and significant sensitization for several days after incision which may be due to excess cytokine production [11]. Also, patients receiving methadone maintenance display mood stabilization at peak concentrations and increased mood disturbance at trough concentrations implying that opioids may directly affect mood states [12–17]. In the future, more patients will be presenting to surgery on opioids. Research indicates that postoperative pain management in these patients will be challenging [18–21].

We conducted an analysis of the data from a previously reported [22, 23] prospective, observational study of opioid cessation after 5 discrete surgical procedures (thoracotomy, total hip replacement, total knee replacement, mastectomy, and lumpectomy). Of 20 variables, only preoperative elevated depressive symptoms, self-perceived susceptibility to addiction, and legitimate preoperative opioid use were independent predictors of delayed opioid cessation after surgery [22].

The aim of this cross-sectional cohort study was to further compare this at-risk population of patients taking opioids prior to surgery to their opioid naïve counterparts. In addition, this study also compared patients taking legitimate, prescribed or illicit, nonprescribed opioids prior to surgery to opioid naïve patients in order to further characterize the distinction between patients with medical and nonmedical use of opioids prior to surgery.

## 2. Materials and Methods

Subjects were recruited between January 2007 and April 2009. Exclusion criteria were any mental incapacity or language barrier precluding completion of assessments. The Stanford University IRB approved the entire protocol, and all participants provided written informed consent.

### 2.1. Measures of Pain and Opioid Use

Prior to surgery, the Brief Pain Inventory (BPI) [24] was administered twice to capture pain limited to the future surgical site, as well as pain elsewhere on the body. Preoperative opioid use was categorized as “legitimate preoperative opioid use” (subjects reported taking opioids directly prescribed to them for pain), “illicit preoperative opioid use” (subjects reported taking opioids not directly prescribed to them), or “any preoperative opioid use” (all subjects from either of the two previous categories).

### 2.2. Measures of Substance Use, Abuse, and Addiction

The Screener and Opioid Assessment for Patients with Pain-Revised (SOAPP-R) was administered to assess the risk of opioid misuse [25]. The self-perceived susceptibility to addiction measure was administered as a response to the question “How likely do you think it is that you will develop an addiction problem from pain medication you take after surgery?” Subjects chose from 4 responses: (1) “not at all”; (2) “unlikely”; (3) “somewhat likely”; or (4) “very likely.” The Addiction Severity Index Drug and Alcohol Use section was used to assess lifetime and previous 30-day use of illicit substances and alcohol [26, 27].

### 2.3. Sleep

The Pittsburgh Sleep Quality Index (PSQI) is a self-rated questionnaire assessing sleep disturbances over the past month [28]. 19 items are administered to generate 7 subscores of subjective sleep quality, sleep latency, sleep duration, habitual sleep efficiency, sleep disturbances, use of sleeping medication, and daytime dysfunction. A global PSQI score was calculated as the sum of these subscores [28].

### 2.4. Psychological Measures

The Beck Depression Inventory-II (BDI-II) [29], Primary Care Posttraumatic Stress Disorder Screen [30], Anxiety-Sensitivity Index [31], and Fear of Pain Questionnaire [32] were administered to assess depressive symptoms, PTSD symptoms, somatic fear and anxiety, and fear of pain, respectively. All baseline questionnaires were assessed between 1 and 2 weeks prior to surgery.

### 2.5. Statistical Analyses

SAS version 9.3 (SAS Institute, Inc., Cary, NC) was used for all analyses. A *t*-test was used to compare all numerical variables, and Chi-square or Fisher's exact test was used to compare all categorical variables. Any preoperative opioid use was analyzed as a distinct dependent variable in the primary logistic regression analyses. As subanalyses, legitimate and illicit preoperative opioid use were then analyzed as distinct dependent variables using separate logistic regression analyses. For each of the subanalyses, those patients reporting both legitimate and illicit preoperative opioid use were excluded in order to directly compare each opioid-using subgroup to their opioid naïve counterparts. SAS PROC LOGISTIC was used for logistic regression, calculation of odds ratios, and model building. The linearity assumption of all significant predictors was met by plotting a linear relationship of the independent variable to the log odds.

Given the paucity of data regarding preoperative characteristics associated with legitimate or illicit preoperative opioid use, we were unable to construct a multivariate model of preselected variables a priori. Instead, all variables found to be significant in univariate analysis were considered for inclusion in the multivariate model. Subsequent multivariate model selection was accomplished by comparing the results of automated stepwise, backward, and forward variable-selection algorithms in order to account for potential errors in model building, sensitivity of the results to the specific model building algorithm, and order effects and to determine optimal model fit.

## 3. Results

Table 1 shows patient characteristics comparing preoperative opioid users to their opioid naïve counterparts. One hundred seven of 134 patients approached for inclusion agreed to participate in the study and provided data on preoperative opioid use.  Table 2 shows patient characteristics comparing illicit and legitimate preoperative opioid users. Of the entire cohort, 8 patients reported both legitimate and illicit preoperative opioid use.

### 3.1. Factors Associated with Preoperative Opioid Use

As shown in Table 1, preoperative opioid users had elevated levels of baseline pain at sites other than their surgical site, elevated SOAPP-R scores, elevated depressive symptoms, and worse sleep. Of note, global PSQI scores >5 are associated with poor sleep quality which would apply to both the preoperative opioid users and the opioid naïve group [28]. Of the entire patient cohort, 65 patients (61%) had a global PSQI score >5. Specifically, 56% (*n* = 45) of the preoperative opioid naïve patients and 74% (*n* = 20) of the preoperative opioid users reported poor sleep quality.

Table 3 shows the results of univariate logistic regression of preoperative variables associated with preoperative opioid use. Of all the variables, only the BDI-II score, SOAPP-R score, and global PSQI score were significantly associated with preoperative opioid use.  Table 4 shows the final multivariate logistic regression model reflecting the adjusted OR for SOAPP-R score. Every 9-point increase in the SOAPP-R score (interquartile range in this cohort) is associated with 2.37 (95% CI 1.29–4.32) increased odds of presenting with preoperative opioid use (*p* = 0.0005). Previous research has reported a cutoff score of 18 or higher to identify patients at high risk for aberrant medication-related behavior [33]. Using these criteria, 21 patients (20%) of the entire cohort had a positive SOAPP-R score. Specifically, 16% (*n* = 13) of the preoperative opioid naïve patients and 30% (*n* = 8) of the preoperative opioid users reported positive SOAPP-R scores.

### 3.2. Factors Associated with Legitimate Preoperative Opioid Use

Table 5 shows the results of univariate logistic regression of preoperative variables associated with legitimate preoperative opioid use. Of all the variables, only the global PSQI score, baseline pain at the future surgical site, and baseline pain elsewhere over the body were significant.  Table 6 shows the final multivariate logistic regression model reflecting the adjusted OR for baseline pain at the future surgical site. Every 4-point increase in baseline pain at the surgical site (interquartile range in this cohort) is associated with 2.85 (95% CI 1.12–7.27) increased odds of presenting with legitimate preoperative opioid use (*p* = 0.03). Also, the global PSQI score was a confounder of the relationship between baseline pain and legitimate opioid use. In the final model, every 7-point increase in the global PSQI score (interquartile range in this cohort) is associated with 2.82 (95% CI 0.98–8.12) increased odds of presenting with legitimate preoperative opioid use.

### 3.3. Factors Associated with Illicit Preoperative Opioid Use

Table 7 shows the results of univariate logistic regression of preoperative variables associated with illicit preoperative opioid use stratified by surgery type. Of all the variables, BDI-II score and SOAPP-R score were significantly associated with illicit preoperative opioid use.  Table 8 shows the final multivariate logistic regression model reflecting the adjusted OR for SOAPP-R score. Every 9-point increase in the SOAPP-R score (interquartile range in this cohort) is associated with 3.02 (95% CI 1.36–6.70) increased odds of presenting with illicit preoperative opioid use (*p* = 0.007).

## 4. Discussion

The main purpose of this study was to identify preoperative factors associated with preoperative opioid use. Of all the preoperative variables examined, only an elevated SOAPP-R score was significantly associated with preoperative opioid use in the final multivariate model. In addition, legitimate and illicit preoperative opioid users were compared to opioid naïve patients as subanalyses. Of all the preoperative variables, baseline pain at the future surgical site was associated with legitimate opioid use, and poor sleep was a confounder of this association. As those patients taking prescribed opioids for pain were classified as legitimate opioid users, we would expect to see an association between elevated levels of pain and legitimate opioid use. Also, an elevated SOAPP-R score was associated with illicit preoperative opioid use. Thus, elevated SOAPP-R scores in illicit preoperative opioid users are potentially driving the association in the entire opioid-using cohort. An important note is that the original study from which this data was drawn was not powered for the present analyses [22]. As the global PSQI score and BDI-II score were both significantly associated with any preoperative opioid use in univariate analyses, it is possible that these associations may be just as important as the SOAPP-R in relation to preoperative opioid use. These findings should be considered in future studies.

The SOAPP-R is a self-reported measure of the risk potential for aberrant medication behavior amongst people with chronic pain [34]. The SOAPP-R has been validated as a screening tool in this population and intended for administration to patients being considered for long-term opioid therapy [34]. Amongst a cross-validation sample for the SOAPP-R, 33.5% of patients had a score of 18 or higher, which was reported as a cutoff score with 80% sensitivity and 52% specificity in identifying patients at risk for aberrant drug-related behavior. In other words, at this cutoff score, the SOAPP-R has a higher chance of identifying false-positives due to this lower specificity. Nonetheless, the SOAPP-R has demonstrated superiority over other self-report measures including the Pain Medication Questionnaire and Opioid Risk Tool to predict discharge from opioid treatment and the presence of aberrant drug-related behavior [35].

Use of the SOAPP-R in the preoperative setting has never been previously reported. Of interest, 30% of the preoperative opioid users and only 16% of the preoperative opioid naïve patients had a positive SOAPP-R score. Furthermore, 24% of legitimate opioid users and 43% of illicit opioid users had a positive SOAPP-R score. These findings are concerning in the context of research showing prolonged postoperative opioid use in patients taking opioids prior to surgery [2–5, 7, 22]. Although this study did not distinguish whether or not patients were already receiving chronic opioid therapy, our results suggest that the positive SOAPP-R score may indicate a potential for aberrant medication behaviors and opioid misuse in the postoperative setting with prolonged opioid use. This is especially likely, as elevated SOAPP-R scores were associated with preoperative self-reported illicit opioid use. In a cohort of chronic pain patients continuing opioid therapy for 12 weeks, a SOAPP-R score greater than or equal to 18 was predictive of a Current Opioid Misuse Measure (COMM) greater than or equal to 9 [36]. The COMM asks patients to describe how they are currently using their medication to identify current opioid misuse. A score of 9 or higher on the COMM has 94% sensitivity and 73% specificity to identify opioid misuse amongst patients with persistent pain [37]. In our present study, we cannot make any assumptions whether or not elevated SOAPP-R scores precede initiation of either legitimate or illicit opioid use. However, the SOAPP-R may be a valuable screening tool in the perioperative setting to identify patients taking preoperative opioids, who are at risk for opioid misuse. Future research should focus on whether elevated preoperative SOAPP-R scores are predictive of elevated postoperative COMM scores.

The global PSQI score was positive (>5) in 61% of our entire patient cohort indicating pervasive poor self-reported sleep quality. While 56% of opioid naïve patients had a positive score, the global PSQI score was positive in 74% of all preoperative opioid users, 81% of legitimate opioid users, and 71% of illicit opioid users, respectively. In addition, the global PSQI score was a confounder of the relationship between baseline pain at the future surgical site and preoperative legitimate opioid use. Although research has established an association between chronic pain and sleep disorders [38], much of the literature examining the effects of chronic opioid therapy on sleep involves patients receiving opioid replacement therapy. Methadone maintenance patients have a high prevalence of sleep disturbances as measured through elevated PSQI scores [39], worse daytime function, and increased subjective daytime somnolence. Less attention has focused on patients with chronic pain, who are also using prescription opioids. In a cross-sectional study of VA patients in a single VA medical center, patients with chronic pain prescribed opioids were more likely to have a sleep apnea diagnosis, as well as an elevated PSQI global score. Opioid dose was also a significant predictor of impaired sleep latency and global PSQI score. In our study, patients reporting legitimate opioid use may have had more consistent opioid exposure resulting in a higher proportion of elevated PSQI scores. In patients with chronic pain receiving chronic opioid therapy, opioid dose is associated with unrefreshing sleep and worse sleep maintenance. Also, use of long-acting opioids is associated with increased napping [40]. In the perioperative setting, screening for poor sleep quality amongst prescription opioid users may be particularly important given the increased and prolonged opioid requirements in the postoperative period through recovery. As a dose-dependent relationship between opioid use, central sleep apneas, and ataxic breathing exists, patients reporting elevated global PSQI scores may be at increased risk for respiratory depression [41]. Research is needed to examine the association between preoperative elevated global PSQI scores and objective measures of impaired sleep in the postoperative setting.

Although depressive symptoms were not associated with preoperative opioid use in any of the final multivariate models, our study found that elevated depressive symptoms were associated with any preoperative opioid use and illicit opioid use in univariate analyses. Previous research has demonstrated an association between nonmedical prescription opioid use with panic, depressive, and social phobic/agoraphobic symptoms [42]. Of note, 57% of the preoperative illicit opioid users in our study also reported legitimate opioid use. In a cohort of primary care patients with chronic pain, Posttraumatic Stress Disorder was a factor associated with prescription drug use disorder [43]. Although we are unable to make assumptions regarding the chronicity of opioid use in our surgical cohort, an association exists between long-term prescription opioid use, affective disorders, and substance abuse. Increased rates of substance abuse and depression exist in long-term prescription opioid users compared to nonusers with chronic pain, and pain intensity does not predict treatment with opioids versus nonopioid analgesics [44]. Other research shows that depression and anxiety contribute to substance use disorders amongst long-term opioid users [45]. As more than half of the illicit opioid users in our cohort were also receiving legitimately prescribed opioids, it is possible that elevated depressive symptoms may have led to nonmedical prescription opioid use.

Similarly, psychological comorbidities are common amongst patients with prescribed opioids for chronic noncancer pain. For example, patients with chronic pain who have psychological comorbidity and higher pain severity ratings are more likely to present on opioids to a tertiary pain clinic [46]. Likewise, veterans with prescribed high doses of opioids (greater than or equal to 180 morphine equivalents daily for 90 days) are more likely to have the highest rates of medical, psychiatric, and substance use disorders (major depressive disorder, Posttraumatic Stress Disorder, anxiety disorders, sleep disorders, or a substance use disorder) [47]. Higher opioid doses are associated with higher levels of patient-reported opioid-related psychosocial problems (loss of interest in activities, trouble concentrating, feeling slowed down and sluggish, feeling depressed or anxious, interference with activities, and decreased alertness), opioid control concerns (being worried about opioid dependence, wanting to stop or cut down use of opioids, and reporting that family or friends think the patient may be addicted to opioids), and depressive symptoms and are more likely to be clinically depressing [48].

Although our study did not capture the chronicity or quantity of preoperative opioid use in our surgical cohort, our previously reported findings demonstrated that an elevated preoperative BDI-II score is an independent predictor of delayed opioid cessation after surgery [22]. Therefore, preoperative assessment of depressive symptoms may have two important benefits: (1) to identify patients who may be using illicit opioids and (2) to identify patients at risk for prolonged opioid use after surgery. Furthermore, testosterone deficiency is a less commonly discussed adverse effect of prescription opioid use. Particularly, testosterone serves a function in endogenous opioid activity through facilitation of opioid receptor binding, activation of dopamine and norepinephrine activity, and maintenance of blood-brain barrier transport [49]. Clinically, decreased testosterone can result in impaired pain control, depression, anxiety, weight gain, decreased muscle mass, reduced quality of life, osteoporosis, fatigue, sleep disturbances, irregular menses, reduced libido, erectile dysfunction, and hot flashes [49, 50]. This opioid-induced androgen deficiency may play a key role in precipitating pain sensitivity and mood disturbances. Future work to preoperatively diagnose opioid-induced hypogonadism and to correlate this endocrinopathy to preoperative mood disturbances and increased pain sensitivity may pinpoint an underlying mechanism for our findings, as well as an important future intervention target.

## 5. Conclusions

Opioid users presenting for surgery have elevated SOAPP-R scores relative to their opioid naïve counterparts. As preoperative opioid users are exposed to surgical injury and escalated and prolonged opioid use in the postoperative period, these elevated SOAPP-R scores may also indicate the potential for opioid misuse. Notably, a high proportion of the entire surgical cohort reported poor sleep quality through elevated global PSQI scores. Future research is needed to further examine objective measures of impaired sleep in the postoperative period.

## Figures and Tables

**Table 1:** Patient characteristics.

	No preoperative opioid use	Any preoperative opioid use	*p* value
Preoperative characteristic (SD)^a^			
Patients (*n*)	80	27	
Age (y)	58 (13)	56 (14)	0.48
Male gender (*n*)	28% (22)	37% (10)	0.35
Baseline pain other than surgical site (0–10)	2.04 (2.45)	3.78 (2.58)	0.002
Baseline pain at surgical site (0–10)	1.58 (2.42)	3.44 (3.03)	0.002
Self-Perceived Risk of Addiction	1.27 (0.55)	1.44 (0.58)	0.15
History of Addiction Treatment (*n*)	4% (3)	4% (1)	1
SOAPP-R score	11.91 (5.74)	19.26 (12.57)	0.006
Used tobacco regularly (lifetime) (*n*)	23% (18)	41% (11)	0.07
Beck Depression Inventory-II score	7.75 (6.11)	14.65 (12.49)	0.01
Anxiety Sensitivity Index Score	14.54 (10.55)	19.48 (15.81)	0.14
Fear of Pain Score	70.12 (22.53)	74.19 (21.92)	0.42
Primary Care PTSD Screen Score	0.49 (1.04)	0.88 (1.45)	0.21
PSQI Total Score	6.96 (3.69)	9.92 (4.67)	0.002
Surgery type (*n*)			0.02
Thoracotomy	28% (22)	19% (5)	
Total knee replacement	14% (11)	30% (8)	
Total hip replacement	19% (15)	37% (10)	
Radical mastectomy	26% (21)	15% (4)	
Lumpectomy	14% (11)	0% (0)	

SOAPP-R, Screener and Opioid Assessment for Patients with Pain-Revised; PTSD, Posttraumatic Stress Disorder; PSQI, Pittsburgh Sleep Quality Index.

^a^Values are presented as means and SD except where noted otherwise.

**Table 2:** Patient characteristics amongst opioid use subgroups^a^.

	Illicit preoperative opioid use	Legitimate preoperative opioid use
Preoperative characteristic (SD)^b^		
Patients (*n*)	14	21
Age (y)	52 (17)	57 (12)
Male gender (*n*)	43% (6)	43% (9)
Baseline pain other than surgical site (0–10)	3.79 (2.58)	3.76 (2.81)
Baseline pain at surgical site (0–10)	3.36 (3.43)	3.62 (2.64)
Self-Perceived Risk of Addiction	1.57 (0.65)	1.43 (0.60)
History of Addiction Treatment (*n*)	7% (1)	5% (1)
SOAPP-R score	23.00 (16.03)	18.43 (11.74)
Used tobacco regularly (lifetime) (*n*)	57% (8)	38% (8)
Beck Depression Inventory-II score	19.92 (15.28)	13.35 (9.98)
Anxiety Sensitivity Index Score	22.71 (18.31)	18.52 (14.17)
Fear of Pain Score	74.29 (25.88)	72.10 (22.11)
Primary Care PTSD Screen Score	1.15 (1.57)	0.85 (1.46)
PSQI Total Score	9.58 (4.08)	10.55 (4.56)
Surgery type (*n*)		
Thoracotomy	21% (3)	19% (4)
Total knee replacement	29% (4)	29% (6)
Total hip replacement	29% (4)	48% (10)
Radical mastectomy	21% (3)	5% (1)
Lumpectomy	0% (0)	0% (0)

SOAPP-R, Screener and Opioid Assessment for Patients with Pain-Revised; PTSD, Posttraumatic Stress Disorder; PSQI, Pittsburgh Sleep Quality Index.

^a^Eight patients reported both illicit and legitimate preoperative opioid use.

^b^Values are presented as means and SD except where noted otherwise.

**Table 3:** Univariate analysis of preoperative variables associated with any preoperative opioid use.

	OR (95% CI)	*p* value
Beck Depression Inventory-II score	1.08 (1.02–1.15)	0.001
SOAPP-R score	1.10 (1.03–1.18)	0.005
PSQI Total Score	1.14 (1.01–1.28)	0.04
Primary Care PTSD Screen Score	1.48 (0.99–2.20)	0.06
Used tobacco regularly (lifetime)	2.40 (0.90–6.38)	0.08
Self-Perceived Risk of Addiction	1.91 (0.87–4.16)	0.11
Baseline pain other than surgical site (0–10)	1.16 (0.95–1.41)	0.15
Anxiety Sensitivity Index Score	1.03 (0.99–1.06)	0.19
Age (y)	0.98 (0.94–1.01)	0.21
Baseline pain at surgical site (0–10)	1.14 (0.92–1.42)	0.24
Fear of Pain Score	1.01 (0.99–1.03)	0.37
Male gender	0.86 (0.31–2.37)	0.77
History of Addiction Treatment	1.04 (0.10–11.39)	0.97

SOAPP-R, Screener and Opioid Assessment for Patients with Pain-Revised; PTSD, Posttraumatic Stress Disorder; PSQI, Pittsburgh Sleep Quality Index.

**Table 4:** Multivariate analysis of preoperative variables associated with any preoperative opioid use.

	Adjusted OR (95% CI)	*p* value
SOAPP-R Score	2.37 (1.29–4.32)	0.0005

Adjusted OR represents every 9-point increase in the SOAPP-R (Screener and Opioid Assessment for Patients with Pain-Revised) score, which is the interquartile range.

**Table 5:** Univariate analysis of preoperative variables associated with legitimate preoperative opioid use.

	OR (95% CI)	*p* value
PSQI Total Score	1.20 (1.04–1.39)	0.01
Baseline pain at surgical site (0–10)	1.33 (1.07–1.65)	0.01
Baseline pain other than surgical site (0–10)	1.28 (1.03–1.59)	0.03

PSQI, Pittsburgh Sleep Quality Index.

**Table 6:** Multivariate analysis of preoperative variables associated with legitimate preoperative opioid use.

	Adjusted OR	*p* value
	(95% CI)	
Baseline pain at surgical site (0–10)	2.85 (1.12–7.27)	0.03
PSQI Total Score	2.82 (0.98–8.12)	0.06

Adjusted OR represents every 4-point increase in the baseline pain score and every 7-point increase in the global PSQI (Pittsburgh Sleep Quality Index) score, which is the interquartile range.

**Table 7:** Univariate analysis of preoperative variables associated with illicit preoperative opioid use.

	OR (95% CI)	*p* value
SOAPP-R score	1.13 (1.04–1.24)	0.007
Beck Depression Inventory-II score	1.11 (1.02–1.20)	0.02

SOAPP-R, Screener and Opioid Assessment for Patients with Pain-Revised.

**Table 8:** Multivariate analysis of preoperative variables associated with illicit preoperative opioid use.

	Adjusted OR (95% CI)	*p* value
SOAPP-R score	3.02 (1.36–6.70)	0.007

Adjusted OR represents every 9-point increase in the SOAPP-R (Screener and Opioid Assessment for Patients with Pain-Revised) score, which is the interquartile range.
